# *“The car is my extra legs”* – Experiences of outdoor mobility amongst immigrants in Sweden with late effects of polio

**DOI:** 10.1371/journal.pone.0224685

**Published:** 2019-10-31

**Authors:** Helena Selander, Iolanda Santos Tavares Silva, Felicia Kjellgren, Katharina S. Sunnerhagen

**Affiliations:** 1 Rehabilitation Medicine, Department of Clinical Neuroscience, Sahlgrenska Academy, University of Gothenburg, Sweden; 2 The Swedish National Road and Transport Research Institute (VTI), Gothenburg, Sweden; 3 Department of Occupational Therapy and Physiotherapy, The Sahlgrenska University Hospital, Gothenburg, Sweden; Chinese Academy of Medical Sciences and Peking Union Medical College, CHINA

## Abstract

**Background:**

The aim of the study was to describe the experience of outdoor mobility among immigrants with late effects of polio living in Sweden. There is a need to understand more about this young group of persons since they often have problems with mobility and gait, but they may also face participation restrictions due to issues associated with integration into a new community and culture.

**Method:**

A total of 14 young immigrants with late effects of polio participated and were interviewed individually. The study used a qualitative method to explore personal experiences and the interviews were analyzed through an inductive approach, using qualitative content analysis.

**Results:**

The analysis led to a major theme; s*elf-image and acceptance*, that comprised a changeable process and experiences of cultural, social, and gender-specific barriers, but also of environmental and personal factors that impacted their outdoor mobility. By using a car, the participants felt they could come across as normal which also increased their self-esteem.

**Conclusions:**

Independent mobility is a major enabler for ongoing employment and being able to use a car increases the chances for integration into society for young immigrants with late effects of polio. Public transport is not considered to be adequate or efficient enough due to the participants’ mobility impairments, but driving can prevent involuntary isolation and facilitate participation. A car can increase quality of life but may also be a facilitator for work and reduce the demand for societal support.

## Introduction

Although polio outbreaks have been eliminated in most parts of the world, many people (12–20 million) still live with the sequelae of polio [1]. Symptoms such as renewed muscle weakness, fatigue, and pain in muscles and joints, as well as cold intolerance may occur many years after acute polio [2, 3]. The symptoms may be related to the original polio infection or be indirectly consequence of post-polio syndrome (PPS). While polio survivors may not fulfil the criteria for PPS diagnosis, many still live with sequelae of polio. These symptoms have been described as “the late effects of polio” [4, 5]. It is estimated that between 15 000 and 20 000 people are living with sequelae associated with polio in Sweden [6]. Polio survivors who are native to Sweden are often around 65 years old, since vaccinations were introduced in the late 1950s [6]. However, the number of young people living with the late effects of polio has increased due to adoption and immigration [7]. The number of people born outside of and living in Sweden has increased; in 2018 the proportion was 19%, compared to 11% in year 2000 [8]. With increasing immigration from countries where polio is still endemic or has only recently disappeared, the Swedish healthcare system faces a new challenge [9,10]. In order to provide the best assistance and treatment it is important to understand more about how the late effects of polio affect these patients in their daily lives [11,12]. For example, they may have impaired outdoor mobility, such as problems with walking, climbing stairs, and using both public and other transport modes, which significantly limits the mobility of these individuals and thereby their participation in the community [13–15].

In health science and rehabilitation, “mobility” as a concept is about moving by changing body position or location, or by transferring from one place to another, by carrying (moving or manipulating objects), by walking, running, or by climbing, and by using various forms of transportation [16]. Outdoor mobility in the local community can be accomplished by walking, using a wheelchair, using public transport or driving one’s own car [17]. Furthermore, the ability to move about in your own community enables participation in several leisure-time and social activities [18]. In the industrialized world, driving is often seen as the most convenient and practical way to travel. For many people, driving is often taken for granted and some may even be reluctant to use public transport [19]. However, for people with disabilities, this may not be a matter of choice [20].

Many activities in daily living such as attending medical appointments and social activities require some sort of transport [21]. Reduced outdoor mobility can have an impact on employment prospects [22], lead to depression and hamper many activities of daily living [20]. The Swedish government’s policy entails a well-established tradition proclaiming every citizen’s equal right to mobility, independence, and quality of life [23]. In Sweden the healthcare system is responsible for providing mobility aids to counteract the effect of disability. Moreover, national regulations require that many transport modes, e.g., buses and rail systems should be accessible for people with disabilities, including wheelchair users [23]. However, there are still many people with mobility impairments, such as people with late effects of polio, who are unable to walk and reach a bus stop, commuter train or metro station, or are unable to board [20, 24] even if they get there. According to Swedish disability legislation a person with a disability having ‘significant mobility difficulties’ and who cannot travel by regular public transport, may be entitled to the Special Transport Service (STS). However, travelling must be carefully planned since the STS (minibus or taxi) must be ordered at least one day in advance which makes it difficult to take any spontaneous journeys.

The late effects of polio cause significant limitations to mobility and thereby participation in the community. The younger, immigrant population of persons with late effects of polio may also face participation restrictions due to the additional requirement of integration into a new culture and community [25, 26]. Road users in Sweden who were born elsewhere are obviously not a homogeneous group. However, there may be cultural norms, how long they've lived in Sweden, language and socio-economical factors that could impact transportation and traffic safety [27]. There is a need to understand more about how the late effects of polio affect this particular group of persons coming to Sweden. Therefore, the aim of this study was to describe the experience of outdoor mobility among immigrants with late effects of polio living in Sweden.

## Material and methods

### Participants

Participants were identified from a previous, quantitative study [20] of patients with late effects of polio who were born outside the Nordic countries. Participants from that study were identified from The Polio Clinic at Sahlgrenska University Hospital in Gothenburg, Sweden. The Polio Clinic is a facility which offers rehabilitation for people with late effects of polio by a multidisciplinary team. People are referred to the clinic due to new symptoms and data from their first visit are entered in a database (based on the Swedish unique personal identification number). To be included in the study, the participants had to be of working age, born outside the Nordic region but able to communicate in Swedish and have been diagnosed with late effects of polio. Participants from the quantitative study (n = 74) were invited to participate in the present qualitative study [20]. A total of 14 individuals gave their written consent to further participate and were contacted by the third author (FK), a female medical student at the time of the data collection.

### Design and procedure

An inductive qualitative design was used for the study, the qualitative approach is often used for data based on narratives and observations [28, 29]. The interviews took place at the homes of the respondents or at the Polio Clinic, depending on what was most practical for the respondents. Some of the questions were open-ended and the participants expressed their thoughts and experiences of outdoor mobility, such as perceived mobility barriers, mobility aids, transport modes they used, experiences of longer journeys, their driver's license and driving. The interviews were performed by the third author (FK) and audio-recorded and transcribed verbatim, with identifying data removed. The study follows the COREQ criteria [30].

### Analyses

The data analyses of the transcribed interviews were carried out according to a method described by Graneheim and Lundman [31]. Qualitative content analysis is suitable for a variety of data, enabling interpretation at various depths and has been mainly used within nursing research and education [31]. The analyses were done in collaboration between the first and the second authors, both holding a PhD in Occupational Therapy, and with broad experience from rehabilitation research and clinical rehabilitation of patients with mobility impairments. Initially, the transcript was read individually by the first and second author, respectively, in order to obtain a general understanding of the studied phenomena. In the next step the text was searched for meaning units containing aspects related to the experience of outdoor mobility and driving. The meaning units were condensed to codes and the codes were furthermore analyzed into categories by comparison of similarities and differences of the codes. Each category was analyzed and main categories with subcategories merged, for examples see Table 1. The categories and subcategories were compared with the initial interview data during the analysis. Throughout the analysis process the first and second authors discussed and reflected on the findings together. Based on the text as a whole, the categories and subcategories and the authors understanding of the underlying latent meanings a theme was formulated; *self-image and acceptance*.

**Table 1:** Examples illustrating the coding procedure.

Meaning units	Condensed meaning units	Subcategory	Category
So like I often say, I can walk 50 meters but in that time I can have fallen over 5 times. I don’t take that sort of risk.	I can walk 50 meters but could have fallen. I don’t take that risk.	Emotional reactions	Decrease in mobility function
I can go without food, but not without my car. That’s how important it is! I see the car as my legs. If I don’t have a car, then I’m handicapped	I can go without food but not my car. I see the car as my legs.	Independence and freedom	A car as an enabling mobility aid

### Establishing trustworthiness

Following Granheim and Lundman [31], strategies to increase credibility and trustworthiness of data were used in the present study. To increase the credibility of the data collection and reduce external influences during data collection, the interviews were carried out in an environment known and chosen by the participants and a decision was made to not to use an interpreter. Language barriers were resolved by checking the interpretations with the respondents during the interviews. The design made it possible to examine the research topic in depth and not limited the content, the allowed the participants to express their experiences. The participants were considered representative of the immigrant population in Sweden living with late effects of polio comprising a heterogenous group with regard to their degree of disability, age, land of origin, work conditions, marital status and family. Since credibility deals with how well categories and themes cover data the results were compared to the data during the analysis. Reflexive strategies and critical considerations of the findings are necessary to handle the authors preconceptions and interpretations of the findings [31]. In this study critical consideration consisted on reflection on the authors’ personal and professional experiences. Furthermore, the results were discussed with co-researchers and polio experts.

### Research ethics

The study follows the Helsinki declaration and was approved by the Regional Ethics Committee in Gothenburg, Sweden in 2016 (Dnr: 674–16). Prior to participation, all participants were provided with information about the study procedures and were informed that they could discontinue their participation at any point without prejudice. All participants gave written informed consent.

## Results

The participants were all born outside the Nordic region; 1 in Europe, 2 in Africa, 9 in Asia and 2 in South America. The age of the 14 participants (7 women and 7 men) ranged from 23 to 57 years (mean age 43 years). The key demographics of the participants are outlined in Table 2. All participants had some degree of walking impairments and different types of mobility aids were reported, such as crutches and wheelchairs. At the time of the interviews, 9 were drivers, 1 was a learner driver and 4 did not drive.

**Table 2:** Participants characteristics (n = 14).

*Participant No*.	Age	Gender/driver	Mobility aids outdoors	Working status
1	42	Female driver	Crutches, powered wheelchair	Disability pension
2	55	Male driver	Cane, lower leg bandage	Disability pension
3	57	Male nondriver	Manual wheelchair	Disability pension
4	32	Female nondriver	Crutches, electric scooter	Unemployed
5	25	Male, candidate driver	Manual wheelchair, electric scooter	Disability pension
6	39	Male driver	Manual wheelchair	Working
7	56	Female driver	Powered wheelchair	Unemployed
8	35	Female nondriver	-	Working
9	54	Male driver	Cane	Working
10	54	Male driver	Cane	Working
11	40	Female driver	Crutches, electric scooter	Working
12	23	Female nondriver	-	Working
13	46	Female driver	Crutches, manual wheelchair	Unemployed
14	48	Male driver	Cane, whole leg bandage	Disability pension

**Fig 1:**
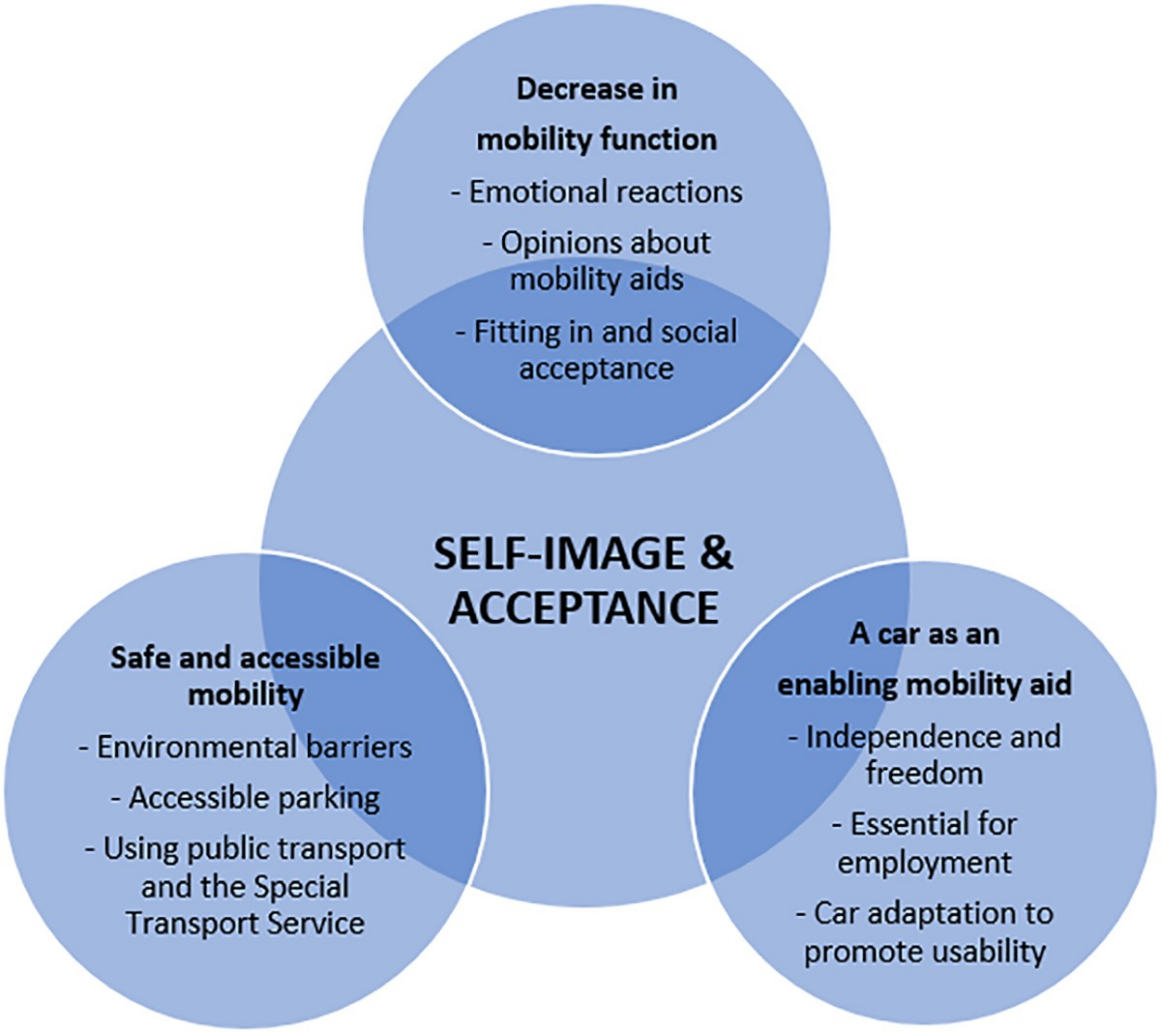
The theme “Self-image & acceptance” comprised an acceptance process affecting self image. There are, three categories and sub-categories influencing the participants’ experiences.

A major theme was identified from the interviews that described the participants’ self-image and acceptance, that was an ongoing process to their changes in their life, e.g., the onset of polio, renewed muscle weakness, emigrating to a new country and culture. Moreover, three categories comprise description of their experiences of outdoor mobility; 1) *Decrease in mobility function*, 2) *Safe and accessible mobility*, 3) *A car as an enabling mobility aid* (Fig 1). The categories and subcategories are illustrated with quotes from the participants.

### Decrease in mobility function

Compared to when they were younger several of the participants felt progressively worse and experienced new or increased symptoms. Due to their post-polio syndrome, they experienced increased problems that affected their functional ability and participation in daily life. This has led to different emotional reactions and affected their view of the need for mobility aids, but also how they view and accept themselves when relating to important others.

*Now that I’m getting a little older I’m noticing that I don’t have the same capacity to walk as I did before*. *I am struggling because my whole body is tired*, *I have to rest*, *I have to sleep because my body tells me it is time to stop*. *I just can’t do what I did before*. *(Participant no 14)*

*It’s often the way with those of us with polio injuries*, *our brains want to do much more than our bodies and that’s the thing that is frustrating that isn’t fun*. *(Participant no 1)*

#### Emotional reactions

Some participants described that they have accepted their impaired functional ability but still struggling with feeling inadequate and lacking confidence. They could not be like everybody else and had to face that life did not turn to be how they expected it to be. These participants caught acute polio as children, but the new symptoms were harder to accept.

*I have lived with this my entire life*, *I don’t know of anything else*, *but I am very worried about how things are now and that I’m going to get worse and that scares me*, *that I’m going to get weaker*. *You end up giving up and feeling down*. *(Participant no 8)*

Several of the participants reported feeling uncomfortable or embarrassed when people looked at them. Other participants experienced that other people did not always understand their difficulties as their impairment was not always "visible", for instance, their need for a seat on the bus due to balance problems. Moreover, the fear of and past experiences with falling [over] often restricted participants in their movement outdoors.

*So like I often say*, *I can walk 50 metres but in that time I can have fallen over 5 times*. *I don’t take that sort of risk*. *(Participant no 1)*

Several participants also struggled with pain which affected their mood and caused negative emotions. While many had learned to live with the pain, they were concerned about the changes that happened to them and how it would be in the future. For example, one of the participants was not able to work anymore and felt rejected from society and wanted more support from the community.

#### Opinions about mobility aids

In Sweden, mobility aids are in general provided free of charge for those who have difficulty walking or are unable to walk. Several of the participants used different kinds of mobility aids outdoors which increased their participation and independence in daily activities, such as crutches or powered wheelchairs. Two of the participants managed without these aids. Several of the participants experienced considerable benefits from mobility aids and thought that these aids were a necessity to be able to be move outdoors. Some of the participants described their mobility aids as a way to facilitate their own participation in everyday activities. For example, thanks to the electric scooter a participant said that she was able to go to the grocery store.

*I have to say*, *if I didn’t have my Permobil (powered wheelchair) I would have been more restricted*. *(Participant no 1)*

However, the participants experienced both positive and negative emotions concerning their mobility aids. While some of the participants were satisfied with their mobility aids, other participants found it harder to accept and come to terms with their impairment and need to use mobility aids.

*I do actually have a crutch*, *but I rarely use it*. *I try to avoid using it as much as I can*. *I leave it in the car if I don’t absolutely have to use it*. *It’s a bit embarrassing*. *(Participant no 10)*

Some participants had developed excuses as to why their mobility aids were not useful, to provide rational explanations for not using them. One participant expressed that the mobility aid was cumbersome to use when moving outdoors or when using different transport modes. Another participant describe that the mobility aid was hidden in their car, and the “disability parking permit” was also hidden there.

-*can’t really imagine a life where I can’t walk independently*, *where I need assistive devices*, *wheelchairs*, *walking frames*. *I don’t see that in my future*, *I refuse to*. *(Participant no 8)*

#### Fitting in and gaining social acceptance

The ability to move around in one’s community enables participation in work, social, cultural and self-care activities, but such mobility may also provide a sense of belonging in society. However, some of the participants avoided taking part in activities as they were too demanding for them. Others had withdrawn from socializing with family or friends and expressed feelings of isolation when they were unable to move outdoors.

*I end up isolated*, *because of course they call and want me to go out with them*, *but I can’t*. *I want to do everything they do*, *but…*. *I can’t*. *(Participant no 4)*

Moreover, there were also emotions about other people’s opinions or expectations. Some of the participants expressed a strong desire to fit in with others but were afraid they were not good enough.

*You could say that you want to be like everyone else*. *It’s not even a competition really*, *but…you often try to be even better than them*. *But you don’t actually have the same capacity*, *and yet you work so hard to try and find solutions*. *(Participant no 10)*

Some cultural differences were raised regarding the view on disability, and on people with disabilities. One participant neither felt accepted by the country of birth nor by family members, but with a “new” life in Sweden both self-image and self-esteem had improved.

*Being handicapped*, *and married off and at the same time poor*, *it’s really awful…Not even your own parents care about you*. *When you’re poor*, *you don’t think about kids who are handicapped…Coming to Sweden felt like an opportunity for me to just be myself*, *without pretending to be something else*. *Here you are loved for who you are*. *(Participant no 12)*

### Safe and accessible mobility outdoors

The participants experienced different kinds of outdoor barriers; environmental barriers when moving in the community, such as limited access to parking lots–although parking specifically adapted for disabled drivers is required by law. Additionally, they emphasized the importance of access to public transportation and the Special Transport Service.

*“Is there a lift/elevator*?*” “No*, *there isn’t*. *It is unfortunately not accessible (for those with a handicap)” which means that you feel excluded in society and you end up feeling bad because of it*. *(Participant no 4)*

#### Environmental barriers

There were several environmental barriers in the outdoor environment when moving outdoors, such as walking on uneven ground, walking up or down a slope and access to buildings. A common limitation among the participants was going up or down stairs. Stairs were also a common barrier when accessing tram stops, the trams themselves, entrances to shops or restaurants etc. Furthermore, cobblestones are common in the Gothenburg area, which caused problems for the wheelchair users.

*In the city there are too many of those small stones*, *what are they called*?*…*. *cobblestones on the streets*! *Yes*, *cobblestones and grit*. *It’s impossible*! *(Participant no 6)*

Several of the participant described how they had fallen and hurt themselves. They often relied on their “healthy leg”, but the fear of losing balance was so substantial that they avoided going outdoors. The experience of several falls on slippery roads, especially during wintertime in Sweden, added their mobility impairments. Snow and ice made it difficult to use a wheelchair or other mobility aids, but the cold also made them feel weaker and more affected.

*If we are talking about winter and snow*, *it’s pretty much screwed*. *I can’t go*, *if there’s snow on the asphalt then I won’t get very far*. *I can’t roll (the assistive device) over it*. *(Participant no 6)*

#### Accessible parking

The “disability parking permit” was another key factor in order to be able to reach a final destination, for example the rehabilitation clinic. An ordinary parking lot could be impossible for people with wheelchairs as they needed extra space to get in or out of a car.

*- but that’s one of the things that I don’t even dare to think about*, *what it would be like without the parking permit*. *If I hadn’t had that I wouldn’t be the person I am today*. *I don’t even think I would have done half the things I’ve done*. *(Participant no 10)*

However, there was often frustration about the number of parking lots outside hospitals, stations and shopping malls. They were often taken and several times they had experiences of people without mobility impairments using them.

*Extremely grateful that you can park for free (when it exists) but unfortunately there are lots of people who use our parking spots…At my last job*, *we made a funny sign that said “if you are going to take my parking spot*, *you might as well take my handicap as well” and that says quite a bit*. *(Participant no 1)*

#### Using public transport and the special transport service

Most of the participants expressed difficulties regarding access to public transport. Although, riding the bus was possible for some of the participants, many experienced difficulties along the way, such as getting on/off the bus or the bus stopped too far away from the curb. Some of the participants had not used public transport for many years due to uncertainty about the accessibility at a specific bus stop.

*I like the new low trams and you can travel on those*, *but the old ones with stairs*, *it’s hard and some doors you need to be able to hold open yourself to get on*, *those are really hard*. *I don’t even take those ones if I’m in a hurry*. *(Participant no 12)*

However, a specific bus stop was not always the main problem. Instead, the participants expressed numerous barriers that they could encounter along the way–from their home to the final destination. This caused too many uncertainties about using public transport, which made them choose to travel by car instead. Most of the participants had positive experiences of assistance from other passengers or staff on the specific tram or bus. However, some of the respondents had had too many negative experiences in the past. Due to their dependence and need of help from other people, they avoided public transport.

*It’s hard to get on and off trams and trains*, *and then you still need the car*. *(Participant no 2)*

The Special Transportation Service (STS) should be able to make community access possible and facilitate participation. However, the participants experienced limited flexibility when using the STS, which made it difficult to make spontaneous trips. They also described the STS as unreliable as they had experienced many delays, and even that the car did not turn up at all. Moreover, some of the participants had experienced delays when commuting to work caused by the STS and expressed the opinion that the STS was a barrier to being able to work full-time. The STS’s requirement to travel with other passengers was also a negative factor when working.

### A car as an enabling mobility aid

Several of the respondents described using a car as a prerequisite to manage daily activities but a car also made them independent in both leisure and work time. The results also indicate the need of vehicle adaptation for safe driving and car access. A car was often described as a mobility aid, but it also symbolized competence and being able to move around in the community like other people, i.e., an accepted mobility aid.

*There are crutches*, *and I have an electric scooter and then I have the car*. *(Participant no 1)*

#### Independence and freedom

A car symbolizes autonomy in everyday life; the importance of deciding your own trips, not having to ask for help or having to wait for the STS. A car also enables longer trips when participants’ mobility function was decreased due to muscle weakness or pain. With a car they were able to carry out activities without other people’s assistance, such as shopping, collecting their children or meeting friends in town. All those activities would have been impossible without a car. The participants repeatedly highlighted the importance of the car for their independence. One participant clearly stated that the car in the participant’s possession was an important mobility aid; for other participants it was about identity and several participants reported feeling “normal or like everybody else” when driving.

*For me the car is extra legs*. *(Participant no 11)*

I *can go without food*, *but not without my car*. *That’s how important it is*! *“I see the car as my legs*. *If I don’t have a car*, *then I’m handicapped”*. *(Participant no 10)*

#### Essential for employment

A number of participants did not have a driver’s license but saw it as a major and important goal for the future. Those who were also unemployed thought a driver's license would increase their opportunity to enter the labor market or had experience of not being able to accept a job due to the distance. There were several examples among the participants when a car had been crucial for getting a specific job. For those with a driver's license, it was outlined that transportation to their workplace would have been difficult without a driver's license and a car. One reason was that public transportation was not available at all for commuting to the workplace or did not run late enough in the evenings or at night.

*Without a car I would work less and I*. *Oh…*. *I wouldn’t work 8 hours and I would work fewer days a week*. *(Participant no 6)*

#### Car adaptation to promote usability

The first step to be able to drive a car was being able to move independently to the driver’s seat and being able to transport and store mobility aids in the car. Most of the drivers were positive about vehicle adaptations as these enable independent and safe use of the car. However, for some of the participants it was harder to accept their mobility impairment and the need for car adaptation.

*I have been very stubborn and sad…I don’t want to have an adapted car*, *I want to be able to just jump in the car whenever*. *(Participant no 8)*

*It’s that thing about feeling like a normal person again*. *It’s really important for us who have polio and are young that everything doesn’t always have to be adapted. (Participant no 1)*

There was a certain frustration regarding car adaptation that did not work as intended and also regarding limitations in financial support in the form of grants or subsidies for vehicle adaptation from the Swedish government. Some participants, who were not eligible for the vehicle adaptation grants, stated they could not afford to pay for vehicle adaptation themselves.

*But we have to do something to make it work*, *so someone or my brother has to lift me in and out of the car*, *that’s not good for their back either*. *(Participant no 5)*

## Discussion

The objective of this study was to describe the experience of outdoor mobility among immigrants with late effects of polio living in Sweden. Unsurprisingly, every participant reported some kind of outdoor mobility problem which additionally affected their self-image and acceptance. For example, the opinions participants had about their mobility aids were made evident by their emotional reactions to them. They felt both positively and negatively about them, but also felt as though they were constantly struggling to fit in and be accepted, regardless of their use of mobility aids. Increasing problems with mobility and gait are common among people with late effects of polio [32] and restrictions in mobility related activities have been previously reported [15]. Although this group of people most often have mobility limitations, only a few studies have previously focused on outdoor mobility and/or transportation [14, 33].

People with late effects of polio might require different kinds of mobility aids for longer distances, due to loss of strength, endurance, or due to pain. It has previously been reported how important mobility aids are for individual wellbeing, independence, social activities and transportation [34]. Despite the benefits gained from the mobility devices, the participants perception of their mobility aids was that they were both facilitators and barriers. Their perception and their acceptance of the specific mobility aid varied and was related to how far they had come in their acceptance process. For example, the use of mobility aids made both their disability and their decreased mobility function visible to others, something they experienced as harmful to their self-image. Similar perception was found in a recent study regarding mobility, where powered wheelchair users (different diagnoses) perceived the aid as an enabler for independence and activities but also that it impacted on their identity and self-esteem [35]. Thus, support from others in the social environment is important for the process of acceptance of the use of mobility aids [35, 36].

The outdoor environment and its accessibility are of vast importance for participation and activity [37]. In a previous study among persons with late effects of polio, a fear of falling was one factor that reduced mobility and impacted on walking activities such as climbing stairs or going shopping [14]. From the present study, it can be concluded that walking limitations and perceived risk of falling had a large effect on the outdoor mobility of several participants. The participants also expressed fear of falling due to previous experience with falling and losing balance, even when equipped with leg braces or crutches. Repeated falls made some participants stay indoors. Moreover, the cold Swedish winters were often reported as a huge challenge for the participants. Snow and ice during winter could prevent the use of their mobility aids outdoors. Among the participants in another study with immigrants living with late effects of polio, the limitations related to the winter conditions were expressed as difficult to overcome and these limitations were shown to be related to feelings of hopelessness [7]. Not being able to move outside your home without assistance from another person represents a major restriction of personal autonomy and freedom [20].

Research about migration shows that migration causes restrictions in all areas of occupational performance [38]. Mobility is no exception and in the present study there are a number of different factors to consider that affect the participants’ outdoor mobility. One such factor for consideration is culture, the participants’ cultural and ethnic affiliation as well as the values in their new home, Sweden. How disability is seen in Sweden was raised in the present study as a positive factor that improved both self-image and self-esteem for the participants and may be considered as a cultural factor of importance for outdoor mobility amongst immigrants in Sweden. This was in contrast to the view on disability in the country of birth for some participants. This result shows the importance of regarding cultural issues with caution, since cultural influences cannot be traced only to the participants’ ethnic and cultural background but also to the society in which they live [7]. Similar results have been emphasized in previous studies about immigrants [7]. Moreover, an important issue to consider is the migration process, where immigrants’ experiences have been described as characterized by loss of everyday security and self-esteem which affected daily occupations, such as mobility and participation, in a complex way [39–41]. Additionally, reduced outdoor mobility for people living with late effects of polio may lead to depression and restrict activities of daily living [20]. These factors, migration and late effects of polio, interact in our study participants making obvious the importance of having a wide perspective when focusing in the intersection of disability and social factors [42].

A goal in traffic planning and urban public transport is to enable all people to travel but the requirements among people with disability are not always covered and they have different preferences depending on their personal characteristics [23]. Although public transport is often accessible, the participants in the study experienced numerous barriers, both with and without mobility aids. Even though the actual public transport vehicle may be accessible, either the station itself or streets and roads leading to it may not be accessible. For example, some environmental barriers were sidewalks with cobblestones, which are common in the Gothenburg area. Thus, using public transport does not only include the actual ride, but a chain of activities, such as planning the trip, walking to the bus/tram, buying tickets and getting on/off the bus/tram [43].

Persons who have a disability preventing them from effortlessly using public transportation are eligible for a car allowance from the Swedish Social Insurance Agency. Drivers with physical impairments may need some sort of adaptation (including automatic gear shifts) such as hand controls to operate the accelerator and brake, or a spinner knob for steering. Although drivers with physical impairments have this need, research is limited or not up to date [15, 20]. Several of the participants in the present study reported a need for vehicle modification to enhance their functions while driving, due to muscle weakness, fatigue and/or pain. It is increasingly important to raise the awareness of the value of adapted cars for people with mobility difficulties, such as people with late effects of polio. The results from the present study should be of interest to those who work with rehabilitation, but also to those who provide and finance transport facilities such as the Swedish Social Insurance Agency. Our study shows that adaptation grants may make it easier for persons with late effects of polio, and others with mobility difficulties, to move independently and freely outdoors. Adaptation grants may also facilitate better/any job prospects and the ability of persons with late effects of polio to engage more fully in family and community activities through increasing access to a well regarded and often used mobility aid, a car.

To use different kinds of transport modes, traffic rules and knowledge should be a part of the integration process into Swedish society. In this study, five men and four women were active drivers. However, it has been previously shown that those born outside Sweden have a lower rate of both having a driver's license and owning a car [44]. Especially for women born outside Sweden, of whom only 20% have a driver's license, vs 45% of men born outside Sweden [44]. For people with physical impairments, driving and owning a vehicle is often the “key” to independent mobility and an enabler for other forms of participation in society [16, 35]. A car enables mobility and access to essential services and social activities [45]. A category that emerged in interviews with the participants was the importance of a car in their lives in order to maintain an active lifestyle with friends and family. Furthermore, by using a car, the participants felt less different from people without disabilities, which also increased their own self-esteem. The participants repeatedly expressed that the car was a facilitator for work, which confirms what has previously been reported [22]. Driving is often important economically for an individual, as many need a car to get to their workplace or they drive a car in a professional capacity. The participants in the present study stated that without access to a car they would not be able to work in rural or suburban areas or would have to limit their working day to fewer hours.

### Strengths and limitations

This study is one of few in this area and is a continuation of a previous study on outdoor mobility for immigrants in Sweden living with late effects of polio. A strength of the present study was the use of a qualitative design which made it possible to enable a better understanding of outdoor mobility and the role of cars. Since native Swedish polio survivors are generally over 65 years old the participants in the present study were all individuals with a foreign background. The interaction of environmental and personal factors was found to impact their mobility, including cultural, societal norms, and gender-specific barriers. The study was performed in a Swedish context and the participants’ experience of public transport stemming primarily from the Gothenburg area. Furthermore, the Swedish STS may not be comparable across all other cities and likely not across countries. Moreover, the number of years the participants had lived in Sweden is unknown, which is a limitation of the study. However, a study strength was the heterogeneity of the participants, representing nearly 50% women, different degrees of mobility impairments and social backgrounds.

## Conclusions

More work is needed on young immigrants with late effects of polio to identify their mobility needs and find solutions that could minimize barriers and help them to be more independent outdoors. This group in Sweden has often been overlooked in clinical settings and in society generally. It is important to consider the need of care and support for persons with late effects polio as the need for care has been questioned and decision makers (such as health commissioners) believe persons with late effects of polio no longer exist in Sweden. Moreover, they are not prioritized despite needing rehabilitation interventions that could increase their outdoor mobility. Independent mobility is a major enabler for ongoing employment and the ability to access a car can increase their chances for social integration. According to participants in this study, public transport is not adequate or efficient enough for their needs. For young immigrants with late effects of polio, driving can prevent involuntary isolation and facilitate participation. A car may increase quality of life and reduce the demand for societal support.
